# Long-term follow-up of ruxolitinib pre-approval use in acute and chronic graft-versus-host disease

**DOI:** 10.1007/s00277-026-07217-4

**Published:** 2026-08-06

**Authors:** Raphael Loisl, Anna Bauhofer, Alexander Nikoloudis, Robert Milanov, Sigrid Machherndl-Spandl, Veronika Buxhofer-Ausch, Michaela Binder, Petra Hasengruber, Emine Kaynak, Dagmar Wipplinger, Irene Strassl, Olga Saini, Christoph Aichinger, Andreas Petzer, Hildegard Greinix, Daniel Wolff, Johannes Clausen

**Affiliations:** 1 Department of Internal Medicine I: Hematology with Stem Cell Transplantation, Hemostaseology and Medical Oncology, Ordensklinikum Linz – Elisabethinen, Fadingerstraße 1, Linz, 4020 Austria; 2 Medical Faculty, Johannes Kepler University, Linz, Austria; 3 Department of Internal Medicine III, Hematology and Medical Oncology, University Hospital Regensburg, Franz-Josef-Strauß-Allee 11, Regensburg, 93053 Germany; 4 Department of Internal Medicine, Division of Hematology, Medical University of Graz, Graz, Austria

**Keywords:** Ruxolitinib, Hematopoietic stem cell transplantation, Refractory graft versus host disease, Overlap GVHD, Donor lymphocyte infusion (DLI)

## Abstract

Steroid-refractory (SR) acute and chronic graft-versus-host disease (aGVHD/cGVHD) are frequent and serious complications after allogeneic hematopoietic stem cell transplantation. Ruxolitinib (RUX) is approved for the treatment of both conditions. Real world evidence may provide valuable complementary information beyond prospective trials because of significant divergences in study populations, endpoints, treatment strategies, and observation time. We report long-term outcomes of 118 patients with acute (n = 61), classic chronic (n = 43) and overlap chronic GVHD (n = 14) who received RUX within a named-patient-program in the pre-approval era as second-line treatment or beyond. In 22 patients, GVHD occurred after donor lymphocyte infusion (DLI). The cohort included heavily pretreated patients, with 26.2% of aGVHD in 3rd -line or beyond, and 29.8% of cGVHD in 4th -line or beyond. Only 22 patients (18.6%) met all in-/exclusion criteria of the respective pivotal phase III studies. The most frequent incompatibilities were unmet SR criteria, excessive pretreatment, previous DLI, overlap chronic GVHD, and medical/hematological criteria. Overall response rate to RUX was 68.9% in aGVHD, and 66.7% in cGVHD. In all three GVHD types, > 50% of patients were able to discontinue systemic steroids. With a survivors’ median follow up of 60.3 months (range, 25.5–99.3) from RUX initiation, 4-year survival probability was 57.4% in aGVHD, 81.1% in classic cGVHD, and 78.6% in overlap cGVHD. Our findings reveal favorable survival probabilities associated with a long-term steroid-sparing/replacing potential of RUX in treatment-refractory aGVHD and cGVHD, including overlap syndrome and DLI-associated GVHD.

**Supplementary Information:**

The online version contains supplementary material available at 10.1007/s00277-026-07217-4.

## Introduction

Allogeneic hematopoietic stem cell transplantation (alloHSCT) is an established treatment of malignant and non-malignant hematological diseases [1]. One of the most frequent and serious complications is graft-versus-host disease (GVHD), in which the grafted donor immune cells recognize the recipient’s tissues as foreign and subsequently trigger a complex immune response that can result in inflammation, tissue damage, and fibrosis. Depending on the clinical presentation, GVHD is classified as acute or chronic GVHD (aGVHD/cGVHD), with cGVHD manifesting as classic form and overlap with the latter being defined by the simultaneous presence of both, aGVHD and cGVHD features [2–4]. 

Despite advances in GVHD prophylaxis, up to 50% of HSCT recipients develop aGVHD [5]. Systemic corticosteroids are generally used as the first-line treatment for aGVHD, although responses may be short-termed, and approximately 50% of patients require second-line treatment for SR aGVHD, which is associated with a marked reduction in quality of life and mortality [6–8]. While incidence of cGVHD declined from 50% to 40% through the last decade half of the patient fails steroids and require prolonged immunosuppression involving multiple lines of treatment [9]. There has been no established therapy approved by the EMA until the approval of RUX in 2nd line treatment of acute and cGVHD in May 2022 [8, 10]. However, overlap cGVHD and patients with GVHD induced by donor lymphocyte infusion (DLI) were excluded from the pivotal REACH 3 trial [6, 7, 11]. Overlap cGVHD, i.e., the co-occurrence of cGVHD with an inflammatory component, is a particular clinical challenge because of lower response rates and increased treatment related mortality [12–14]. Finally, DLI-induced GVHD may differ from primary GVHD types, and is associated with a relatively high mortality [15–17] Therefore, real-world evidence, covering the above scenarios, may be a valuable complement to the findings derived from the prospective studies. Herein we describe our observations in pre-approval usage dating from 2015 until approval in 2022 focusing on patients who would have been not eligible to be enrolled in the pivotal trials.

## Patients and methods

### Patients

In this single-center retrospective study, all patients who received RUX within a named-patient-program in the pre-approval era as treatment for SR GVHD as a second or subsequent treatment line during May 2015 until September 2022 were included. Baseline characteristics are shown in Table 1. Response was evaluated if a patient received RUX for at least 14 days, or until death, which applied to all 118 patients. Safety was documented for all patients who received at least one dose of RUX. The evaluated patients were classified into three subgroups based on the clinical type of GVHD at RUX initiation (aGVHD, classic cGVHD and overlap cGVHD) according to published criteria [18]. Supportive care was performed according to centre-specific standards.

**Table 1:** Baseline characteristics of RUX study population in aGVHD, classic cGVHD and overlap cGVHD

	Overall	Acute	Classic chronic	Overlap chronic
Patient characteristics				
Number of patients, *n* (%)	118	61	43	14
Recipient age (years) at transplant, Median [range]	55.1 [18.1 to 73.6]	56.8 [22.2 to 70.7]	51.9 [18.1 to 73.5]	55.1 [24.4 to 68.8]
Sex, *n* (%) Male / female	75/43 (64/36)	40/21 (66/34)	24/19 (56/44)	11/3 (79/21)
Recipient CMV serostatus IgG positive, *n* (%)	59 (50)	32 (52)	22 (51)	5 (36)
Primary disease / underlying diagnosis / transplant indication, *n* (%)				
ALL	14 (11.9)	6 (9.8)	7 (16.3)	1 (7.1)
AML	56 (47.5)	30 (49.2)	22 (51.2)	4 (28.6)
MDS/MPN	28 (23.7)	12 (19.7)	8 (18.6)	8 (57.1)
Lymphoma, myeloma, other	20 (16.9)	13 (21.3)	6 (14.0)	1 (7.1)
Disease risk at transplant, *n* (%)				
rDRI low	3 (2.5)	0 (0)	3 (7,0)	0 (0)
rDRI intermediate	77 (65.2)	38 (62.3)	28 (65.1)	11 (78.6)
rDRI high	30 (25.4)	17 (27.9)	11 (25.6)	2 (14.3)
rDRI very high	8 (6.8)	6 (9.8)	1 (2.3)	1 (7.1)
Transplant characteristics / donor type, *n* (%)				
Matched related	31 (26.3)	13 (21.3)	12 (27.9)	6 (42.9)
Matched unrelated	32 (27.1)	11 (18.0)	19 (44.2)	2 (14.3)
MMUD	6 (5.1)	4 (6.6)	2 (4.7)	0 (0)
UCB (unrelated)	1 (0.9)	1 (1.6)	0 (0)	0 (0)
Haploidentical	48 (40.7)	32 (52.5)	10 (23.3)	6 (42.9)
Donor characteristics, *n* (%)				
Sex mismatched female donor	31 (26.3)	14 (23.0)	13 (30.2)	4 (28.6)
Donor age in years, Median [range]	36.9 [19.7 to 67.1]	42.7 [19.7 to 67.1]	33.3 [21.1;64.0]	39.5 [22.5;60.4]
Donor CMV serostatus IgG positive	43 (36.4)	22 (36.1)	16 (37.2)	5 (35.7)
Graft source, *n* (%)				
Bone marrow	9 (7.6)	3 (4.9)	5 (11.6)	1 (7.1)
Peripheral blood stem cells	108 (91.6)	57 (93.4)	38 (88.4)	13 (92.9)
Cord blood	1 (0.9)	1 (1.6)	0 (0)	0 (0)
Conditioning regimen, *n* (%)				
MAC	80 (67.8)	46 (75.4)	23 (53.5)	11 (78.6)
RIC	38 (32.2)	15 (24.6)	20 (46.5)	3 (21.4)
GVHD prophylaxis, *n* (%)				
CSA-MMF	32 (27.1)	13 (20.3)	15 (34.9)	4 (28.6)
CSA-MTX	33 (28.0)	12 (19.7)	17 (39.5)	4 (28.6)
PTCY-TAC-MMF	53 (44.9)	36 (59.0)	11 (25.6)	6 (42.9)
ATLG-based prophylaxis	42 (35.6)	23 (37.7)	16 (37.2)	3 (21.4)
GVHD onset following DLI				
*n* (%)	22 (18.6)	7 (11.5)	12 (27.9)	3 (21.4)
Treatment lines prior to RUX for the respective GVHD type, *n* (%)				
1 (= steroids only)	69 (58.5)	45 (73.8)	15 (34.9)	9 (64.3)
2	27 (22.9)	11 (18.0)	13 (30.2)	3 (21.4)
3	14 (11.9)	4 (6.6)	9 (20.9)	1 (7.1)
4	3 (2.5)	0 (0)	3 (7.0)	0 (0)
5	2 (1.7)	0 (0)	1 (2.3)	1 (7.1)
6	2 (1.7)	1 (1.6)	1 (2.3)	0 (0)
> 6	1 (0.8)	0 (0)	1 (2.3)	0 (0)
Maximum steroid dose any time before RUX initiation for the respective GVHD type in mg methylprednisolone equivalent per kilogram body weight per day				
Median [range]	1.1 [0.0 to 6.7]	1.9 [0.07 to 6.7]	0.8 [0.0 to 4.7]	1.1 [0.08 to 3.2]

Abbreviations: *ALL* acute lymphoblastic leukemia, *AML* acute myeloid leukemia, *MDS* myelodysplastic syndrome, *MPN* myeloproliferative neoplasm, *MMUD* mismatched unrelated donor, *UCB* umbilical cord blood, *MAC* myeloablative conditioning, *RIC* reduced intensity conditioning, *GVHD* graft versus host disease, *CSA* cyclosporine A, *MTX* methotrexate, *PTCY* posttransplant cyclophosphamide, *TAC* tacrolimus, *MMF* mycophenolate mofetil, *ATLG* anti-T-lymphocyte globulin

### Endpoints and definitions

The objective of this study was to evaluate the tolerability and the effectiveness of RUX in treatment refractory aGVHD and cGVHD in a patient population receiving RUX during the pre-approval era on a named-patient use basis. Further, we aim to describe treatment patterns prior to, as well as after the initiation of RUX.

The primary endpoint of the evaluation was best overall response rate (ORR) at any time, defined as the proportion of patients who achieved complete response (CR) or partial response (PR) while on RUX treatment during the observation period.

Since RUX was frequently continued even if an additional treatment line was initiated for the purpose of combination treatment, response was evaluated as best response separately for RUX monotherapy and for the combination treatment. In addition to best ORR at any time, response was evaluated on day 28 and three months for aGVHD, and 3 months and 6 months for cGVHD, and at the last RUX dose in patients who discontinued RUX or last follow up in patients with ongoing RUX treatment, until March 31st, 2023. The evaluation at predetermined timepoints was conducted irrespective of the presence of an additional treatment line in combination with RUX.

Survival was assessed for an extended observation period, with database lock on March 31st, 2025. Overall survival was calculated from the date of first RUX administration until death from any cause or last follow up.

Major secondary endpoints were the latest response while RUX is taken at last follow up, the feasibility of steroid taper, duration of RUX treatment, overall survival, and causes of death after initiation of RUX treatment, cytomegalovirus (CMV) reactivation during RUX and the description and quantification of treatment lines before RUX, while on RUX treatment (in case of combination treatment) and as a substitute for RUX in case of RUX failure.

Grading and staging of aGVHD was defined using the MAGIC criteria, and grading and staging of cGVHD was defined using the 2005 NIH consensus criteria, revised in 2014 [4, 18–23]. 

Response was classified according to published criteria for aGVHD and cGVHD. Response categories were complete response (CR), partial response (PR) and no response (including stable / unchanged or progressive GVHD). Complete remission (CR) is defined as complete response in all organs (= stage 0) and fully resolved symptoms. Partial remission (PR) is defined as a response in at least one organ, without worsening, recurrence, or a new onset of GVHD in any organ, but without fully resolved symptoms. The group of “no response” equals treatment failure and includes all patients with mixed response, stable or progressive disease [24–26]. 

If RUX treatment was discontinued due to lack of efficacy or progression, patients were also reported as “no response/treatment failure”.

Conditioning regimens were classified as myeloablative (MAC) versus reduced intensity (RIC) defined by the Centre of International Blood and Marrow Transplant Research in 2009 [27, 28]. 

The disease stage (disease risk) at the time of transplantation was categorized according to the CIBMTR disease risk index [14].

Treatment lines prior to RUX include all immunosuppressive drugs including steroids and/or calcineurin inhibitors (CNI), which were employed to treat the symptoms of GVHD. This does not include ongoing drugs used for the prophylaxis of GVHD.

Treatment lines added after RUX initiation included all immunosuppressive drugs intended for the treatment of GVHD that were initiated after the first dose of RUX. This encompasses any pharmacological agent introduced in conjunction with RUX during the observation period or employed as an alternative to RUX when its administration was terminated.

Adverse events (AE) occurring during RUX therapy were evaluated according to the Common Terminology Criteria for Adverse Events (CTCAE) Version 5.0, dated November 27, 2017 [29]. As cytopenia was frequently observed prior to the administration of RUX, particularly in aGVHD, hematological adverse effects are classified as treatment-emergent adverse events (TEAE). This implies that the deterioration of preexisting cytopenia is indicated by the grade of worsening.

### Statistical analysis

The study was approved by the Ethics Commission of the Johannes Kepler University (EK Nr: 1117/2022 and 1061/2023). In accordance with the Declaration of Helsinki, all patients provided written informed consent to the treatment, and to data collection and analysis. The data were retrospectively gathered from the electronic patient records of the hospital via the hospital information system. Descriptive statistics were employed to calculate medians and ranges. Overall survival was plotted using a Kaplan-Meier curve, and the survival curves were compared using a log-rank test. To facilitate the presentation of the best response and response at the final follow-up, and visualization of causes of death, a stacked bar plot was created for each GVHD group.

All data were evaluated, processed and analyzed graphically using the statistical software R (R Foundation for Statistical Computing, Vienna, Austria).

## Results

### Patients

A total of 118 patients who developed GVHD following alloHSCT were treated with RUX outside the prospective studies (REACH-2, REACH-3) as a second or subsequent line of therapy at Ordensklinikum Elisabethinen in Linz, Austria between May 2015 and September 2022, and were included in this retrospective data analysis. The treatment response of GVHD and subsequent treatment patterns were evaluated until 31st March 2023 while for survival, an extended follow-up was applied until 31st March 2025.

Table 1 presents the baseline characteristics of patient demographics and transplantation for the overall cohort and the subgroups classified according to the type of GVHD.

Of the total cohort of 118 patients, 61 patients (51.7%) were treated with RUX for aGVHD, 43 patients (36.4%) received RUX for classic cGVHD, and 14 patients (11.9%) were treated with RUX for overlap cGVHD.

In total, 22 (18.6%) of the GVHD episodes investigated occurred following the administration of unscheduled (i.e., either preemptive or therapeutic) donor lymphocyte infusion (DLI) (Table 1). Of the DLI-associated GVHD cases, 7/61 (11.5%) were aGVHD, 12/43 (27.9%) were classic cGVHD and 3/14 (21.4%) were overlap cGVHD.

The median follow-up for survivors of the entire cohort was 60.3 months from RUX initiation (range, 25.5 to 99.3 months).

The study population included patients who had undergone extensive prior treatment, as illustrated in Table 1. Organ staging at RUX initiation is shown in the supplementary table S2, more detailed GVHD characteristics at RUX initiation are illustrated in supplementary table S7.

The median time from transplantation to the commencement of RUX was 7.0 months (range, 0.3–170.2) for the entire cohort. It was 1.5 months (range, 0.3–72.0) for aGVHD, 9.1 months (range, 2.4–64.0) for overlap cGVHD, and 23.4 months (range, 5.5–170.2) for classic cGVHD (Table 2).

**Table 2:** RUX and steroid intake information in aGVHD, classic cGVHD and overlap cGVHD

	Overall	Acute	Classic chronic	Overlap chronic
	118	61	43	14
Time from transplantation to RUX initiation in months				
Median [range]	7.0 [0.3 to 170.2]	1.5 [0.3 to 72.0]	23.4 [5.5 to 170.2]	9.1 [2.4 to 64.0]
Time from start systemic steroid requiring GVHD to RUX initiation in months				
Median [range]	1.2 [0.0 to 165.7]	0.5 [0.0 to 10.4]	7.7 [0.0 to 165.7]	0.7 [0.0 to 38.3]
Topical steroid use at RUX initiation				
Steroid mouthwash Number of patients, n (%)	22 (18.6)	3 (4.9)	13 (30.2)	6 (42.9)
Budesonide capsules usage Number of patients, n (%)	60 (50.8)	51 (83.6)	5 (11.6)	4 (28.6)
Steroid dose over the course for the respective GVHD type, median [range] indicated as mg methylprednisolone per kilogram of body weight per day				
Maximum dose (any time before RUX initiation)	1.1 [0.0 to 6.7]	1.9 [0.07 to 6.7]	0.8 [0.0 to 4.7]	1.1 [0.08 to 3.2]
At RUX initiation	0.7 [0.0 to 2.6]	1.9 [0.0 to 2.3]	0.1 [0.0 to 2.1]	0.2 [0.0 to 2.6]
At end of RUX treatment or last follow up	0.0 [0.0 to 2.7]	0.0 [0.0 to 2.1]	0.0 [0.0 to 0.3]	0.0 [0.0 to 2.7]
RUX dosage in mg, median [range]				
At initiation	10 [5 to 40]	10 [5 to 20]	10 [5 to 40]	10 [5 to 20]
Maximum dosage	16.1 [4 to 40]	20 [7.9 to 30]	15 [4 to 40]	20 [10 to 20]
At last follow up	2.1 [0.0 to 30]	1.4 [0.0 to 20]	5 [0.0 to 30]	1.7 [0.0 to 20]
RUX treatment duration in months				
Median [range]	15.6 [0.1 to 74.4]	6.8 [0.1 to 64.7]	33.9 [2.1 to 74.4]	27.9 [0.2 to 66.2]

The median time from the initial onset of steroid-requiring GVHD to the initiation of RUX was 1.2 months (range, 0–165.7) for the entire cohort. It was 0.5 months (range, 0–10.4) for aGVHD, 0.7 months (range, 0–38.3) for overlap cGVHD, and 7.7 months (range 0–165.7) for classic cGVHD.

Among the patients with classic cGVHD, onset of cGVHD was new onset in 7 (16.3%), quiescent onset in 23 (53.5%) and progressive onset in 13 patients (30.2%).

### Evaluation of REACH-2 and REACH-3 eligibility criteria in the named patient use (NPU) population

To elucidate potential differences between the investigated NPU cohort and the prospectively studied REACH-2 and REACH-3 cohorts, the respective eligibility (i.e., inclusion and exclusion) criteria were reviewed in the NPU cohort. Seven patients met the exclusion criterion of prior study participation including investigational agents for GVHD prevention and treatment.

Reasons for not being eligible for one of the Phase 3 REACH studies are listed in Table 3 summing up to > 100% since most patients were treated outside the enrollment periods and/or had multiple other ineligibilities present. In 58 of the 118 patients (49.2%) RUX NPU was initiated before or after the respective REACH study recruitment period. In addition, particularly in the cGVHD group, a high proportion of patients did not meet the steroid-refractory/-dependency criteria [18, 30, 31]. In the cGVHD cohort (classic + overlap), 61.4% were steroid-dependent (35/57), but in 29.8% (17/57) of cases, steroids alone were not the most recent line of therapy, with patients showing non-responsiveness to additional or subsequent treatments (Table 1). Within the aGVHD cohort, 31.1% (19/61) were steroid-refractory and 16.4% (10/61) were steroid-dependent. In the aGVHD cohort, 28/61 patients met the SR criteria, whereas in the cGVHD cohort, only 8/43 patients fulfilled the SR criteria for the corresponding REACH-trial. Other reasons for ineligibility were excessive pretreatment (≥ 2 lines for aGVHD and ≥ 2–3 lines for cGVHD; 28%), DLI-associated GVHD (18.6%), inappropriate medical or hematological conditions (4.2%), previous relapsed malignancy (11.9%) and overlap cGVHD (11.9%).

**Table 3:** Characterization of the cohort according to in- and exclusion criteria of the respective Phase 3 trial (REACH-2 or REACH-3): reasons for not being eligible-

*n*	Overall	aGVHD	cGVHD (classical & overlap)
	118	61	57 (43 + 14)
Start NPU outside the RCT recruitment period	58 (49.2)	21 (34.4)	37 (64.9)
Reasons for not being eligible for the respective phase 3 trial, *n* (%)			
Prior participation in a study addressing investigational agents for GVHD prevention or treatment	7 (5.9)	2 (3.3)	5 (8.8)
Steroid refractory/dependency criteria not met*	52 (44.1)	23 (37.7)	29 (50.9)
RUX beyond second/third line**	33 (28.0)	16 (26.2)	17 (29.8)
DLI-induced GVHD	22 (18.6)	7 (11.5)	15 (26.3)
Relapse after allogeneic HSCT	14 (11.9)	6 (9.8)	8 (14.0)
Lack of haematological regeneration	5 (4.2)	4 (6.6)	1 (1.8)
Overlap GVHD	14 (11.9)	0 (0)	14 (24.6)
Serious illness or other medical indication for exclusion	11 (9.3)	8 (13.1)	3 (5.3)

Abbreviations: *DLI* donor lymphocyte infusion, *NPU* named patient use, *RCT* randomized controlled trial

*The criteria for steroid-refractory or dependency were only met when RUX was used in the 1st line (aGVHD) or 2nd line (cGVHD) of treatment.

**more than 1 line (steroids) in aGVHD or more than 2 lines (steroids plus other) for cGVHDNumbers sum up to > 100% since several patients met more than one exclusion criterion.

### Treatment details

As illustrated in Table 2, the median initial RUX dose in aGVHD and cGVHD was lower than in the respective REACH-studies but subsequently increased to the recommended dose of 2 × 10 mg daily. Of the 118 patients, a total of 49 (41.5%) received additional therapy after initiation of RUX, including 28/61 (45.9%) in the aGVHD group, 15/43 (34.9%) in the classic cGVHD group, and 5/14 (35.7%) in the overlap GVHD group. The median time to next treatment was 9.5 days for aGVHD, 120 days for classic cGVHD, and 52 days for overlap GVHD.

In 30 patients (25.4%), RUX could be discontinued due to the attainment of complete remission (Table 4), including 22 patients with aGVHD (36.1%), 7 patients with classic cGVHD (16.3%) and 1 patient with overlap cGVHD (7.1%). As expected, the median duration of RUX treatment was shorter in aGVHD than it was in cGVHD (Table 2). Among the patients who were able to discontinue RUX due to complete remission, the median duration of treatment with RUX was 6.8 months (range, 1.9–60.7 months) in the aGVHD group and 24.1 months (range, 4.8–71.9 months) in the cGVHD group (classic + overlap).

**Table 4:** Reasons for RUX discontinuation in aGVHD, classical cGVHD and overlap cGVHD

	Overall	Acute	Classical chronic	Overlap chronic
	118	61	43	14
RUX ongoing at last follow up	49 (41.5)	16 (26.2)	25 (58.1)	8 (57.1)
Overall treatment discontinuation, *n* (%)				
RUX discontinued	69 (58.5)	45 (73.8)	18 (41.9)	6 (42.9)
Reasons for RUX discontinuation, *n* (%)				
CR achieved	30 (25.4)	22 (36.1)	7 (16.3)	1 (7.1)
Toxicity, intolerance	6 (5.1)	4 (6.6)	2 (4.7)	0 (0)
Lack of efficacy, GVHD progression	12 (10.2)	8 (13.1)	3 (7.0)	1 (7.1)
Progression of GVHD with infection	8 (6.8)	4 (6.6)	3 (7.0)	1 (7.1)
Progression of underlying disease with persisting GVHD	2 (1.7)	1 (1.6)	1 (2.3)	0 (0)
Progression of underlying disease without infection or GVHD	7 (5.9)	3 (4.9)	1 (2.3)	3 (21.4)
Infection	4 (3.4)	3 (4.9)	1 (2.3)	0 (0)

Abbreviations: *CR* complete remission

Other reasons for RUX discontinuation included the progression of GVHD due to lack of efficacy (10.2%), the progression of GVHD with coexistent infection (6.8%), the progression of underlying disease (without coexisting infection or GVHD, 5.9%), and toxicity or intolerance (5.1%). Overall treatment discontinuation occurred in 45 of 61 (73.8%) patients with aGVHD, and in 24 of 57 (42.1%) patients with cGVHD, with no difference between classic and overlap cGVHD.

The median dose of steroids at the time of RUX initiation (methylprednisolone equivalent) is described in Table 2. In 23/61 (37.7%) of patients with aGVHD, and in 29/57 (50,9%) of patients with cGVHD (including overlap), the initial steroid doses were too low, and/or steroid treatment duration was too short for meeting the criteria for SR GVHD as required in the pivotal trials by consensus definitions [18]. 

At the reference date (March 31st, 2023), 63.75% (51/80) of surviving patients were steroid-free. The remaining 29 patients received < 7.5 mg daily or even lower non-daily doses. Consequently, all evaluable patients at final follow-up were below Cushing threshold, with the majority being entirely steroid-free. This reduction was observed in all three forms of GVHD.

### Treatment response

**Fig. 1:**
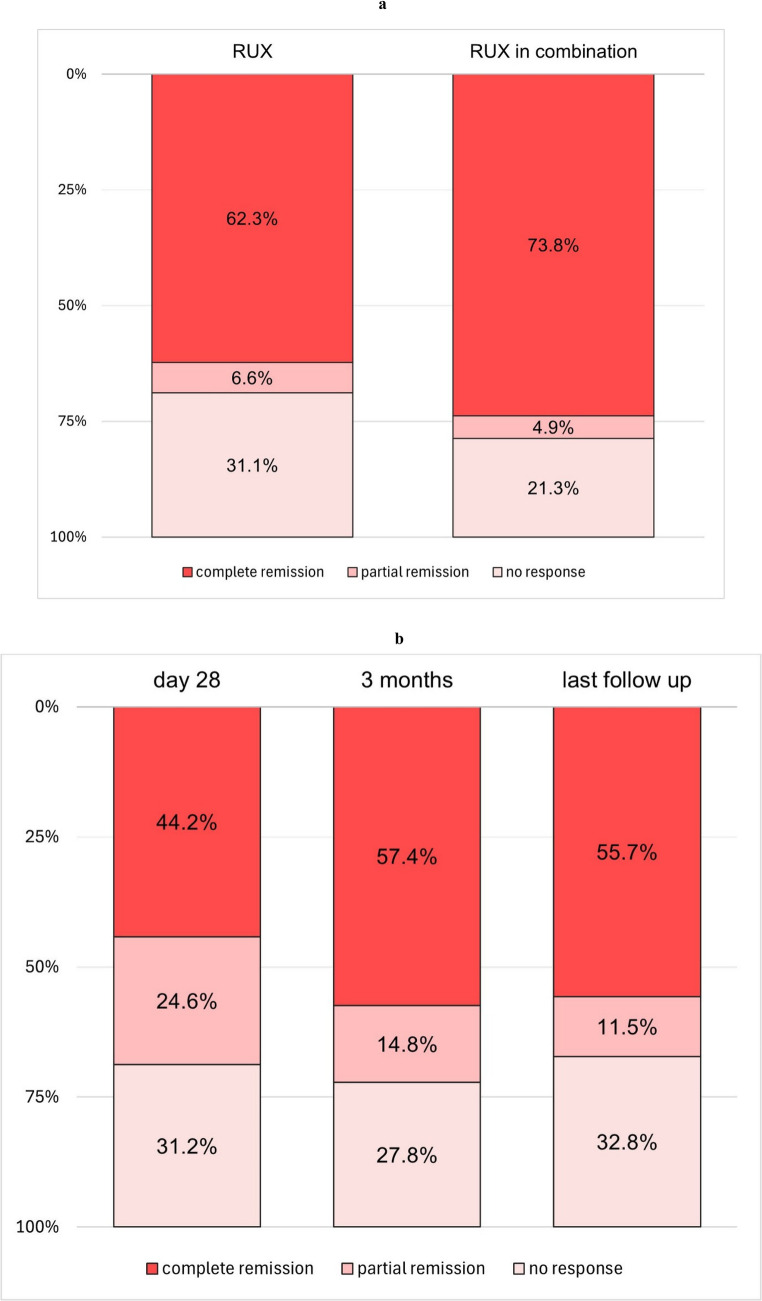
**a** Rates of best response to RUX and RUX combinations in aGVHD. “RUX in combination” is including patients responding to RUX alone and those responding after the addition of another treatment to RUX. **b** Response rates in aGVHD at day 28, after 3 months and last follow-up, respectively. *Response rates were evaluated at defined time points, regardless of whether RUX was used alone or in combination

In patients with aGVHD, an overall response to RUX (at any time, Fig. 1a) was observed in 68.9% (42/61) of patients, with 38 (62.3%) complete remissions (CR) and 4 (6.6%) partial remissions (PR). The median time from the initiation of RUX to the best response in aGVHD was 1.1 months (range, 0,2–6 months). Response on day 28, at 3 months and last follow-up is shown in Fig. 1b.

Due to the robust response rates in all three GVHD types, 69/118 GVHD patients (58.5%) did not require any additional treatment line after RUX (Table 5). However, 49 patients (41.5%) received one or more GVHD treatment lines, either in addition to RUX, or replacing RUX. Table 6 describes individual treatment lines which were added to or substituted for RUX for the treatment of GVHD for the overall cohort and the GVHD type subgroups, respectively.

**Table 5:** Treatment lines added to or substituted for RUX for treatment of aGVHD, classical cGVHD and overlap cGVHD

	overall	acute	classical chronic	overlap chronic
	118	61	43	14
number of added GVHD treatment lines after RUX initiation, *n* (%)				
0	69 (58.5)	33 (54.1)	27 (62.8)	9 (64.3)
1	22 (18.6)	12 (19.7)	6 (14.0)	4 (28.6)
2	12 (10.2)	8 (13.1)	3 (7.0)	1 (7.1)
3 or > 3	15 (12.7)	8 (13.1)	7 (16.3)	0 (0)

**Table 6:** Agents added to or substituted for RUX for treatment of aGVHD, classical cGVHD and overlap cGVHD

*n* (%)	Overall	aGVHD	Classical cGVHD	Overlap cGVHD
	118	61	43	14
Agents added to RUX for treatment of GvHD				
Cyclosporine A	8 (6.8)	1 (1.6)	5 (11.6)	2 (14.3)
Tacrolimus	6 (5.1)	3 (4.9)	2 (4.7)	1 (7.1)
Mycophenolate mofetil	8 (6.8)	5 (8.2)	3 (7.0)	0 (0)
Methotrexate	6 (5.1)	1 (1.6)	5 (11.6)	0 (0)
Sirolimus	3 (2.5)	1 (1.6)	1 (2.3)	1 (7.1)
Anti-thymocyte globulin	1 (0.9)	1 (1.6)	0 (0)	0 (0)
Extracorporeal photopheresis	19 (16.1)	12 (19.7)	6 (14.0)	1 (7.1)
UVA-therapy	1 (0.9)	0 (0)	1 (2.3)	0 (0)
Etanercept	22 (18.6)	18 (29.5)	4 (9.3)	0 (0)
Infliximab	8 (6.8)	8 (13.1)	0 (0)	0 (0)
Rituximab	12 (10.2)	8 (13.1)	4 (9.3)	0 (0)
Tocilizumab	2 (1.7)	2 (3.3)	0 (0)	0 (0)
Abatacept	1 (0.9)	0 (0)	1 (2.3)	0 (0)
Fecal microbiota transplantation	2 (1.7)	2 (3.3)	0 (0)	0 (0)
Imatinib	3 (2.5)	0 (0)	3 (7.0)	0 (0)
Ibrutinib	2 (1.7)	0 (0)	0 (0)	0 (0)
Ixazomib	2 (1.7)	0 (0)	2 (4.7)	0 (0)
Belumosudil	1 (0.9)	0 (0)	1 (2.3)	0 (0)

Abbreviations: *UVA* ultraviolet A

In the aGVHD group, 18 of 61 patients (29.5%) received a new line of treatment without discontinuation of RUX (RUX combination). Among these 18 patients, 5 (27.8%) converted from no response to RUX mono to CR upon RUX combination, and 1 (5.6%) converted from no response to PR upon RUX combination. Two patients converted from PR to CR upon combination treatment. The remaining 11 patients (61.1%) had no improved response to combination treatment, as compared to RUX alone, with 10 of them remaining unresponsive/progressive, and 1 remaining in PR. Overall, the addition of a new line of treatment to ongoing RUX treatment resulted in an improved ORR in aGVHD, reaching 78.7% (48/61) comprising 45 CR (73.8%) and 3 PR (4.9%). Twenty-one (34.4%) of the patients who were treated with RUX for aGVHD subsequently developed classic cGVHD. The Alluvial plots (supplementary fig. S1 and S2) illustrate the transition patterns for aGVHD and classic cGVHD from start of RUX until last follow-up.

**Fig. 2:**
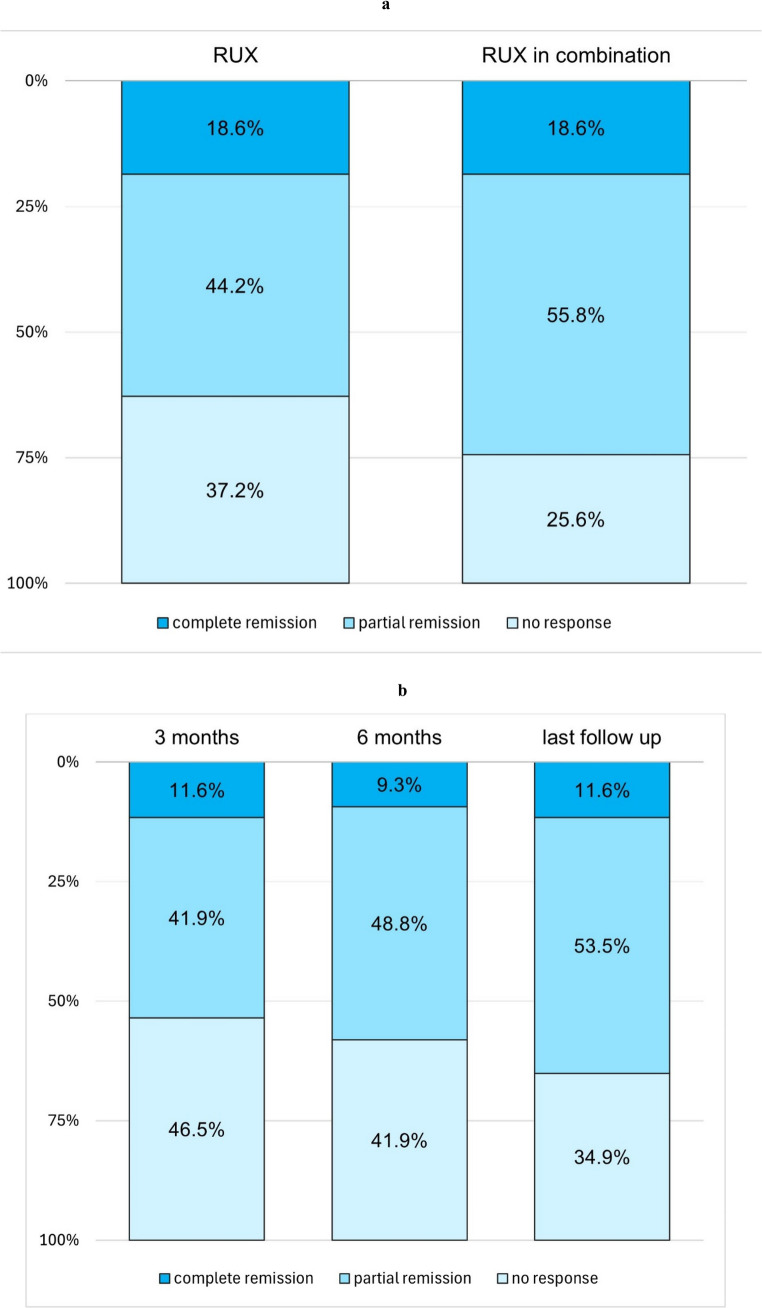
**a** Rates of best response to RUX and RUX combination in classic cGVHD. “RUX in combination” is including patients responding to RUX alone and those responding after the addition of another treatment to RUX. **b** Response rates in classic cGVHD at 3, 6 months and last follow-up, respectively. *Response rates were evaluated at defined time points, regardless of whether RUX was used alone or in combination. *No response= Stable disease, mixed response, progressive disease

In classic cGVHD, the ORR to RUX was 62.8% (27/43), with 8 patients (18.6%) achieving CR and 19 patients (44.2%) achieving a PR as the best response to RUX (Fig. 2a). Among the 16 patients classified as having no response to RUX monotherapy, 3 patients (7.0%) were scored as having mixed response (MR), 12 patients (27.9%) had stable disease (SD), and one patient (2.3%) had progressive disease (PD) despite treatment with RUX. The addition of another agent in combination with RUX resulted in an increase in the ORR to 74.4% (CR 18.6%, PR 55.8%), including patients on RUX monotherapy and RUX combination. In detail, among the 43 patients with classical cGvHD, 10 (23.3%) received a new line of treatment without discontinuation of RUX. Among these 10 patients, one (10%) achieved CR upon combination treatment, and 3 (30%) achieved PR, while the remaining 6 patients (60%) had no additional benefit from combination treatment (4 SD, 1 MR, 1 PD). In the classic cGVHD group, the ORR at the last follow-up was 65.1%, comprising 5 patients (11.6%) in CR and 23 patients (53.5%) who achieved PR. The median time to the best response was 5.5 months (range, 0.4 to 48.4 months). Response evaluation at 3 months, 6 months and last follow up is shown in Fig. 2b.

In patients with overlap cGVHD (*n* = 14), the best ORR to RUX monotherapy was 78.6%, comprising 5 (35.7%) CR and 6 (42.9%) PR (supplementary fig. S3a). In the overlap cGVHD group (*n* = 14), 7 patients (50%) escalated to combination treatment following an incomplete response to RUX monotherapy. While one patient converted from non-response to PR, 6 patients with PR upon RUX monotherapy showed no further improvement. Ultimately, the addition of further agents, the response rate could be improved to 85.7% (5 [35.7%] CR and 7 [50.0%] PR). Two patients (14.3%) exhibited progressive disease during RUX treatment. The median time to the best response was 0.9 months (range, 0.5–19.5). Response over time is shown in the supplementary fig. S3b. At the time of last follow-up, the ORR was 64.3% with 3 (21.4%) CR and 6 PR (42.9%). Three patients died, one due to GVHD, one of infection, and one due to relapse of the underlying malignancy. GVHD treatment at last follow up is shown in the supplementary table S5. Flares of GVHD are illustrated in the supplementary table S3.

### Response in DLI-associated GVHD as compared to primary GVHD

Given the fact that GVHD associated with unscheduled DLI was excluded in both prospective (REACH-2 and REACH-3) trials, we separately assessed the response rates in DLI-induced GVHD and compared them to response rates in non-DLI-induced GVHD (supplementary table S1).

Of the 61 patients in the aGVHD group, 7 patients had DLI-associated GVHD. Among these, four patients (57.1%) achieved CR upon treatment with RUX. Furthermore, upon combination of a new drug with RUX, this rate was increased to 6 out of 7 (85.7%). Similarly, in the non-DLI induced aGVHD group, 34 out of 54 patients (63%) achieved CR after initiation of RUX, and 39 out of 54 achieved CR (72.2%) upon combination of RUX with a new drug.

In DLI-associated cGVHD (15/57, including classic and overlap cGVHD), 3 out of 15 patients (20%) with RUX monotherapy achieved CR. Similarly, in the non-DLI-induced cGVHD group, 10/44 (23%) achieved a CR with RUX, which also could not be improved by combination treatment.

Omong the 22 patients who received RUX for GVHD occurring after preemptive DLI, 4 patients (18.2%) developed clinical relapse during the follow-up period, which ultimately led to their deaths, including 2 patients with DLI associated aGVHD and 2 patients with DLI associated classic cGVHD. By contrast, only 5 out of 96 (5.2%) patients who received RUX for non-DLI associated GVHD experienced disease relapse during the follow-up period.

### Response in intestinal acute GVHD and pulmonary and sclerotic cutaneous cGVHD

In the aGVHD group, 56/61 (91.8%) had aGVHD with lower gastrointestinal (GI) involvement. Among those, 41/56 (73.2%) achieved lower GI CR, 4/56 (7.1%) a lower GI PR, and 11/56 (19.6%%) GI-GVHD did not respond to treatment.

The presence of lung manifestations of cGVHD is associated with a poor prognosis [32]. In our cohort, 15 patients exhibited lung cGVHD (14 classic cGVHD, 1 overlap cGVHD) at the time of RUX initiation, including 6 patients with stage 1 lung involvement, 7 patients with stage 2 and 2 patients with stage 3 (supplementary table S4).

In total, 4/15 patients (26.7%) exhibited an organ-specific response for lung cGVHD, including 2 CR and 2 PR. All those 4 patients had stage 2 lung involvement prior to RUX initiation. Nine patients had SD of lung involvement, including four patients with an initial organ grade 1, three patients with an initial grade 2, and two patients were classified as having grade 3 lung involvement. Two patients with an initial lung disease grade 1 had progressive lung involvement to grade 2 (*n* = 1) and grade 3 (*n* = 1) lung cGVHD, respectively.

Another cGVHD manifestation considered difficult to treat is the sclerotic GVHD of the skin. In our cohort, two out of eight patients with sclerotic skin features exhibited complete resolution of skin involvement cGVHD, corresponding to a CR rate of 25%. In the remaining six patients, the sclerotic features did not respond. But despite the persistence of sclerotic features, the patients reported improvements in skin flexibility and range of motion.

### Overall survival

**Fig. 3:**
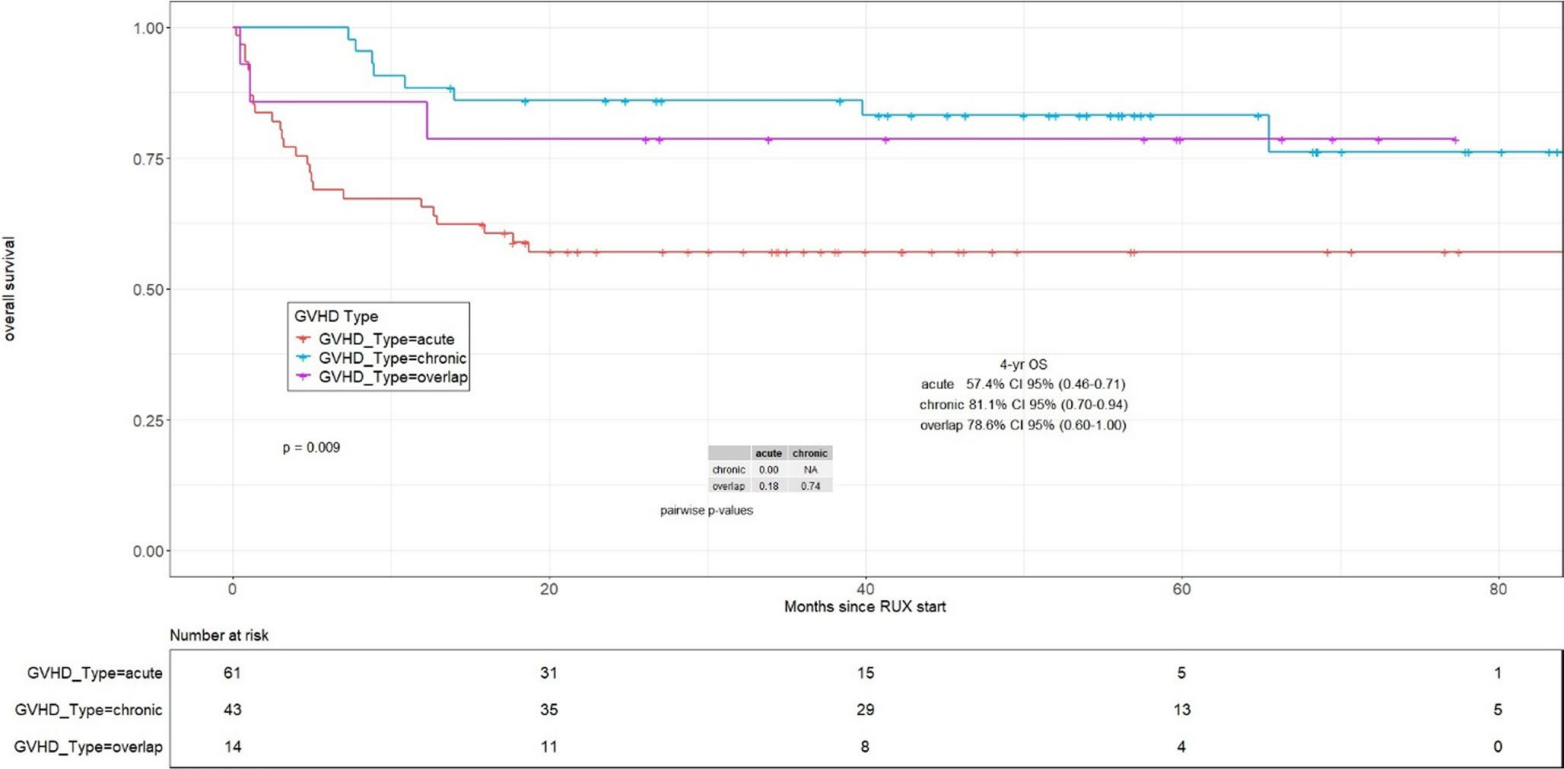
Overall survival from initiation of RUX according to GVHD type

The 4-year OS probabilities from the first day of RUX treatment were 57.4% in acute, 81.1% in classic cGVHD, and 78.6% in overlap cGVHD (Fig. 3). Causes of death are shown in the supplementary table S6.

### Safety and tolerability

The most frequently observed treatment emergent adverse events (TEAE) in the group of aGVHD patients were progressive cytopenia. Since, particularly in aGVHD, blood counts were frequently not fully recovered at the time of RUX onset, toxicities are described as gradual worsening compared to baseline (Table 7).

**Table 7:** Adverse events during RUX treatment in aGVHD, classical cGVHD and overlap cGVHD

*n* (%)	Overall	Acute	Classical chronic	Overlap
	118	61	43	14
Anemia* (grade of worsening in relation baseline)				
Any grade	93 (78.8)	53 (86.9)	30 (69.8)	10 (71.4)
Grade 1	65 (55.0)	38 (62.3)	21 (48.8)	6 (42.9)
Grade 2	24 (20.3)	12 (19.7)	9 (20.9)	3 (21.4)
Grade ¾	4 (3.4)	3 (4.9)	0 (0)	1 (7.1)
Thrombocytopenia* (grade of worsening in relation baseline)				
Any grade	55 (46.6)	41 (67.2)	9 (20.9)	5 (35.7)
Grade 1	27 (22.9)	19 (31.1)	6 (14.0)	2 (14.3)
Grade 2	10 (8.5)	7 (11.5)	1 (2.3)	2 (14.3)
Grade ¾	18 (15.3)	15 (24.6)	2 (4.7)	1 (7.1)
Leukopenia* (grade of worsening in relation baseline)				
Any grade	74 (62.7)	42 (68.9)	19 (44.2)	13 (92.9)
Grade 1	20 (16.9)	11 (18.0)	4 (9.3)	5 (35.7)
Grade 2	26 (22.0)	12 (19.7)	12 (27.9)	2 (14.3)
Grade ¾	28 (23.7)	19 (31.1)	3 (7.0)	6 (42.9)
Infections occurring during RUX treatment				
Any grade	90 (76.3)	47 (77.1)	33 (76.7)	10 (71.4)
Grade ¾	47 (39.8)	30 (49.2)	13 (30.2)	4 (28.6)
Sepsis	15 (12.7)	9 (14.8)	5 (11.6)	1 (7.1)
CMV-reactivation* during RUX treatment among patients at risk				
% of patients at risk**	26/69 (37.7)	22/37 (59.5)	3/26 (11.5)	1/6 (16.7)

*Treatment-emergent adverse events (TEAE), i.e. worsening by CTCAE grade compared to baseline value/ medical history

**CMV seronegative recipients of CMV seronegative grafts were not considered for calculating the reactivation rate

New onset or worsening of anemia was observed in 53 patients (86.9%), of which merely 3 (4.9%) were grade 3–4. A decrease in white blood cells was documented in 42 patients (68.9%), with worsening of leukopenia by 3–4 grades in 19 patients (31.1%). Decreases in platelet counts were observed in 41 patients (67.2%), of which 15 (24.6%) were grade 3–4.

Virtually all patients with refractory aGVHD had posaconazole treatment when RUX was initiated, while patients with cGVHD rarely had azole treatment in parallel with RUX. We did not observe toxicities that were clearly attributable to the combination, although hematotoxicity was indeed higher in aGVHD than in cGVHD. We believe that this finding is inherent to the early postSCT phase and the nature of aGVHD vs. cGVHD itself, rather than related to the combination of RUX and posaconazole.

Infections occurred in 47 out of 61 (77.1%) aGVHD patients, of which 30 (49.2%) were grade 3–4, including 9 patients (14.8%) with sepsis.

During RUX treatment for classic cGVHD, cytopenia was less frequent and less severe compared to RUX treatment of aGVHD (Table 7). Treatment-emergent infections occurred in 33 out of 43 patients with classic cGVHD (76.7%), of which in 13 patients (30.2%) these were grade 3–4. The approximately 5-fold longer median duration of RUX treatment (33.9 months in classical cGVHD, as compared to 6,8 months in aGVHD) and the accordingly longer time at risk needs to be considered.

In overlap cGVHD, blood count changes were less frequent and less severe than in aGVHD. Here, infections were most prominent, occurring in 10 out of 14 patients (71.4%), including grade 3–4 infection in 4 (28.6%) patients.

CMV reactivation occurred in a total of 26 of 69 (37.7%) patients at risk (i.e., donor and/or recipient seropositive). The incidence of CMV reactivation during RUX treatment varied considerably between cohorts: while 59.5% (22/37) of patients at risk in the aGVHD group experienced reactivation, the rates for overlap and classic cGVHD were lower at 16.7% (1/6) and 11.5% (3/26), respectively.

## Discussion

The REACH-2 and REACH-3 studies have shown significant response rates to RUX in SR aGVHD and classic cGVHD, respectively. However, evidence on the efficacy of RUX salvage treatment in overlap cGVHD and in GVHD occurring after interventional (non-scheduled) DLI is sparse, since both conditions were not eligible to the above prospective trials. The present retrospective analysis was performed in a real-world cohort of patients in need of GVHD salvage treatment, including a substantial proportion of patients with overlap cGVHD and patients who developed GVHD after interventional DLI.

Our retrospective analysis confirms the robust responsiveness to RUX in terms of best overall response, response at specified time points, and durability of response. Moreover, we demonstrate a high survival probability after initiation of RUX, despite excessive pretreatment beyond 2nd /3rd line in a proportion of patients. On the other hand, in the real world of RUX as 2nd -line treatment of GVHD, SR criteria have frequently not been awaited as required in the prospective phase III studies [6, 7, 18]. In these patients, the more liberal initiation of RUX salvage treatment in both aGVHD and cGVHD may have contributed to the favorable survival outcomes in our retrospective cohort. In both aGVHD and cGVHD, the response rates and durability of response were at least comparable to those observed in the respective pivotal REACH studies. Although we have primarily assessed the response to RUX itself, we have further followed outcomes in patients who added a new drug to RUX in terms of a combination treatment. While in a prospective phase 3 trial such a scenario is reasonably not permitted and would thus be rated as treatment failure, combining with RUX instead of substituting for RUX may well be common clinical practice. This approach has indeed resulted in an improvement in the best overall response rates both in aGVHD and in cGVHD. Moreover, this way of efficacy assessment seems to be appropriate in the real-world scenario, where subtle nuances of both responsiveness and toxicity may frequently result in the establishment of a two- (or multiple-) drug combination treatment in GVHD, in which dose reductions of individual drugs are sometimes inevitable. The most frequent RUX-based combination treatments included extracorporeal photopheresis in both aGVHD and cGVHD, anti-TNF-treatments in aGVHD, and calcineurin inhibitors in cGVHD. Due to the retrospective (05/2015-03/2023) observation period, fecal microbiota transplantation (FMT) was not yet used frequently in our cohort, but has gained interest since then [33, 34]. Clinical real-world data and retrospective analyses indicate that the combination of RUX and ECP is feasible and associated with encouraging response rates in high-risk patients with steroid-refractory aGVHD and cGVHD, including those with gastrointestinal involvement and advanced disease stages [35–37]. Importantly, combined treatment has been associated with durable disease control and a reduced incidence of cGVHD following aGVHD compared to ruxolitinib alone, without an apparent increase in non-relapse mortality or infectious complications [38, 39]. 

To illustrate the position of RUX in the context of previous and subsequent treatment lines, as well as in the path from a first refractory aGVHD episode to subsequent (quiescent or progressive onset) cGVHD, all treatment lines before and after initiation of RUX were assessed in detail. Particularly in classic cGVHD, a major proportion of the patients (65.1%) had undergone 2 or more treatment lines prior to RUX initiation, due to the onset of cGVHD a long time before the availability of RUX for GVHD treatment. Despite extensive pretreatment in this proportion of patients, response to RUX was considered sufficient, since no additional agent had to be added in conjunction with RUX in most cases (58,5%).

A unique feature of the present study is the inclusion and separate evaluation of DLI-induced GVHD. While in DLI-induced SR aGVHD, response rates were at least comparable to those in primary SR aGVHD, those in DLI-induced cGVHD were numerically slightly lower than in non-DLI induced cases. This may be explained by a more cautious immunosuppressive policy in DLI-induced GVHD, considering the given high risk of relapse of the underlying malignancy, and the resulting acceptance of a certain degree of residual GVHD activity in cGVHD as compared to the scenario of aGVHD. Despite the more cautious use of immunosuppressive therapy, our study nevertheless showed a significantly higher relapse rate in DLI-associated patients treated with RUX compared to non-DLI-associated GVHD patients treated with RUX (18.2% vs. 5.2%). This finding is corroborative of the standard practice of excluding DLI-associated GVHD in prospective studies, mainly because of the high risk of confounding clinical relapses, rather than due to an expected poorer GVHD response rate. Together, our findings suggest the feasibility of RUX salvage treatment in the setting of both DLI-induced aGVHD and cGVHD.

As an additional novel observation, no differences could be identified in response rates comparing classic and overlap cGVHD.

Moreover, difficult-to-treat cGVHD manifestations, such as pulmonary and sclerotic-cutaneous cGVHD, have been separately addressed, both showing promising responses in a proportion of patients.

Regarding gastrointestinal aGVHD, Biavasco et al. reported an organ specific overall response rate (ORR) of 44.5% and a complete response (CR) rate of 13% at day 28 after ruxolitinib initiation in patients with steroid-refractory lower gastrointestinal aGVHD. In contrast, our cohort demonstrated higher response rates in intestinal GVHD. However, it has to be taken into account that the lower GI ORR of 76.8% in our study refers to best overall response and not day 28 response, and is including both responses achieved during RUX monotherapy as well as during RUX in combination [40]. 

An important goal of GVHD salvage therapy is steroid-sparing. In all 3 types of GVHD (aGVHD, classic cGVHD, and overlap cGVHD), we were able to document a steroid termination in more than half of the patients during RUX treatment. The clinical relevance of the high response rates in the investigated cohort is supported by encouraging 4-year OS probabilities from the first day of RUX treatment.

Tolerability of RUX in the present cohort was comparable to prospective studies, with hematologic toxicities being most frequent. Particularly in aGVHD, but also in overlap cGVHD, hematologic toxicity was common during RUX treatment, not only because of preexisting cytopenia, but also because of a higher proportion of patients with worsening blood counts, as compared to patients with cGVHD. These findings are in accordance with those from the prospective trials, which revealed a higher risk for hematological toxicity in aGVHD (REACH-2) compared to cGVHD (REACH-3). Importantly, the above mentioned randomized controlled trials have shown that hematological toxicities are not unique to RUX treatment, but also occurred in the comparator (“best available treatment”) arms of the respective studies [6, 7]. 

Moreover, our study is characterized by an outstandingly long follow-up of up to more than 8 years from RUX initiation, with a median of > 5 years in survivors. This is not only significantly longer than in the prospective studies [6, 7] but also exceeds most other real-world evidence (RWE) cohorts [41–49].

The major limitation of the present study results from the monocentric, retrospective design and the relatively small sample size. However, for an optimal comparability to both, the prospective studies and previously published retrospective analyses, we assessed response rates not only in terms of a best ORR at any time, but also at prespecified time points in accordance with the prospective studies.

In summary, our retrospective study not only confirms the results of the REACH-2 and − 3 studies in a “real-world setting” but also points to a possible benefit of RUX in high-risk GVHD scenarios such as DLI-induced GVHD, overlap cGVHD, and excessively pretreated GVHD, which were excluded from the prospective trials. Given the longest reported follow-up among similar studies so far, we can show encouraging long-term survival rates after RUX initiation in all refractory GVHD subgroups, including specific high-risk situations.

## Supplementary Information

Below is the link to the electronic supplementary material.

Supplementary Material 1 (DOCX 44.2 KB)

Supplementary Material 2 (DOCX 600 KB)

## Data Availability

The datasets generated and/or analyzed during the current study are available from the corresponding author on reasonable request.
