# Supplementary material for: Long-term follow-up of ruxolitinib pre-approval use in acute and chronic graft-versus-host disease
Source: Ann Hematol. 2026 Aug 6;105(8):358. doi: 10.1007/s00277-026-07217-4 (PMC13447468; doi:10.1007/s00277-026-07217-4)
Supplement: Supplementary file 1 [file 277_2026_7217_MOESM1_ESM.docx]

**Supplementary material**

**Table S1** **Response in DLI-induced aGVHD, classical cGVHD and overlap cGVHD compared to non DLI induced GVHD**

| **acute GvHD onset following DLI, n (%)** | | | | | |  |  |  |  |  |
| --- | --- | --- | --- | --- | --- | --- | --- | --- | --- | --- |
| **61 patients** | overall  61 (100) | DLI received 7 (11.5) | | no DLI received 54 (88.5) | |  |  |  |  |  |
| **response reached with** | | **RUX mono** | **RUX ± combi** | **RUX mono** | **RUX ± combi** |  |  |  |  |  |
| **ORR**  (CR + PR) | 48 (78.7) | 5 (71.4) | 7 (100) | 37 (68.5) | 41 (75.9) |  |  |  |  |  |
| CR | 45 (73.8) | 4 (57.1) | 6 (85.7) | 34 (63.0) | 39 (72.2) |  |  |  |  |  |
| PR | 3 (4.9) | 1 (14.3) | 1 (14.3) | 3 (5.6) | 2 (3.7) |  |  |  |  |  |
| **no response** | 13 (21.3) | 2 (28.6) | 0 (0) | 17 (31.5) | 13 (24.1) |  |  |  |  |  |
| **classical chronic GvHD onset following DLI, n (%)** | | | | | |  |  |  |  |  |
| **43 patients** | overall  43 (100) | DLI received 12 (27.9) | | no DLI received 31 (72.1) | |  |  |  |  |  |
| **response reached with** | | **RUX mono** | **RUX ± combi** | **RUX mono** | **RUX ± combi** |  |  |  |  |  |
| **ORR** (CR+PR) | 32 (74.4) | 7 (58.3) | 10 (83.3) | 20 (64.5) | 22 (71.0) |  |  |  |  |  |
| CR | 8 (18.6) | 3 (25.0) | 3 (25.0) | 5 (16.1) | 5 (16.1) |  |  |  |  |  |
| PR | 24 (55.8) | 4 (33.3) | 7 (58.3) | 15 (48.4) | 17 (54.8) |  |  |  |  |  |
| **no response**  (less than PR) | 11 (25.6) | 5 (41.7) | 2 (16.7) | 11 (35.5) | 9 (29.0) |  |  |  |  |  |
| SD | 6 (14) | 3 (25.0) | 0 (0) | 9 (29.0) | 6 (19.4) |  |  |  |  |  |
| MR | 4 (9.3) | 1 (8.3) | 1 (8.3) | 2 (6.5) | 3 (9.7) |  |  |  |  |  |
| PD | 1 (2.3) | 1 (8.3) | 1 (8.3) | 0 (0) | 0 (0) |  |  |  |  |  |
| **overlap chronic GvHD onset following DLI, n (%)** | | | | | |  |  |  |  |  |
| **14 patients** | overall  14 (100) | DLI received  3 (21.4) | | no DLI received  11 (78.6) | |  |  |  |  |  |
| **response reached with** | | **RUX mono** | **RUX combi** | **RUX mono** | **RUX combi** |  |  |  |  |  |
| **ORR** (CR+PR) | 12 (85.7) | 2 (66.7) | 2 (66.7) | 9 (81.8) | 10 (90.9) |  |  |  |  |  |
| CR | 5 (35.7) | 0 (0) | 0 (0) | 5 (45.5) | 5 (45.5) |  |  |  |  |  |
| PR | 7 (50) | 2 (66.7) | 2 (66.7) | 4 (36.4) | 5 (45.5) |  |  |  |  |  |
| **no response**  (less than PR) | 2 (14.3) | 1 (33.3) | 1 (33.3) | 2 (18.2) | 1 (9.1) |  |  |  |  |  |
| SD | 0 (0) | 0 (0) | 0 (0) | 1 (9.1) | 0 (0) |  |  |  |  |  |
| MR | 0 (0) | 0 (0) | 0 (0) | 0 (0) | 0 (0) |  |  |  |  |  |
| PD | 2 (14.3) | 1 (33.3) | 1 (33.3) | 1 (9.1) | 1 (9.1) |  |  |  |  |  |
| Abbreviations: ORR=overall response rate, CR=complete remission, PR=partial remission, SD=stable disease, MR=mixed response, PD=progressive disease | | | | | |  |  |  |  |  |

**Table S2** **Organ staging of aGVHD, classical cGVHD and overlap cGVHD at RUX initiation**

|  | **overall** | **acute** | **classical chronic** | **overlap chronic** |
| --- | --- | --- | --- | --- |
|  | **118** | **61** | **43** | **14** |
| **acute GvHD organ staging at RUX initiation, n (%)** | | | | |
| **no aGvHD** | 44 (37.3) | 0 (0) | 42 | 2 (14.3) |
| **aGvHD upperGI** stage 1 | 34 (28.8) | 31 (50.8) | 0 (0) | 3 (21.4) |
| **aGvHD lowerGI** | 61 (51.7) | 56 (91.8) | 0 (0) | 5 (35.7) |
| loGI stage 1 | 12 (10.2) | 10 (16.4) | 0 (0) | 2 (14.3) |
| loGI stage 2 | 23 (19.5) | 22 (36.1) | 0 (0) | 1 (7.1) |
| loGI stage 3 | 19 (16.1) | 18 (29.5) | 0 (0) | 1 (7.1) |
| loGI stage 4 | 7 (5.9) | 6 (9.8) | 0 (0) | 1 (7.1) |
| **aGvHD skin** | 28 (23.7) | 19 (31.2) | 0 (0) | 9 (64.3) |
| skin stage 1 | 13 (11.0) | 8 (13.1) | 0 (0) | 5 (35.7) |
| skin stage 2 | 6 (5.1) | 4 (6.6) | 0 (0) | 2 (14.3) |
| skin stage 3 | 8 (6.8) | 7 (11.5) | 0 (0) | 1 (7.1) |
| skin stage 4 | 1 (0.9) | 0 (0) | 0 (0) | 1 (7.1) |
| **aGvHD liver** | 2 (1.7) | 2 (3.3) | 0 (0) | 0 (0) |
| liver stage 1 | 0 (0) | 0 (0) | 0 (0) | 0 (0) |
| liver stage 2 | 1 (0.9) | 1 (1.6) | 0 (0) | 0 (0) |
| liver stage 3 | 1 (0.9) | 1 (1.6) | 0 (0) | 0 (0) |
| liver stage 4 | 0 (0) | 0 (0) | 0 (0) | 0 (0) |
| **chronic GvHD organ staging, n (%)** | | | | |
| **no cGvHD** | 61 (51.7) | 61 (100) | 0 (0) | 0 (0) |
| **cGvHD skin** | 26 (22.0) | 0 (0) | 23 (53.5) | 3 (21.4) |
| skin mild | 6 (5.1) | 0 (0) | 5 (11.6) | 1 (7.1) |
| skin moderate | 6 (5.1) | 0 (0) | 5 (11.6) | 1 (7.1) |
| skin severe | 14 (11.9) | 0 (0) | 13 (30.2) | 1 (7.1) |
| **cGvHD GI** | 7 (5.9) | 0 (0) | 7 (16.3) | 0 (0) |
| GI mild | 4 (3.4) | 0 (0) | 4 (9.3) | 0 (0) |
| GI moderate | 3 (2.5) | 0 (0) | 3 (7.0) | 0 (0) |
| GI severe | 0 (0) | 0 (0) | 0 (0) | 0 (0) |
| **cGvHD eyes** | 29 (24.6) | 0 (0) | 22 (51.2) | 7 (50.0) |
| eyes mild | 14 (11.9) | 0 (0) | 10 (23.3) | 4 (28.6) |
| eyes moderate | 11 (9.3) | 0 (0) | 9 (20.9) | 2 (14.3) |
| eyes severe | 4 (3.4) | 0 (0) | 3 (7.0) | 1 (7.1) |
| **cGvHD mouth** | 38 (32.2) | 0 (0) | 28 (65.1) | 10 (71.4) |
| mouth mild | 22 (18.6) | 0 (0) | 15 (34.9) | 7 (50.0) |
| mouth moderate | 16 (13.6) | 0 (0) | 13 (30.2) | 3 (21.4) |
| mouth severe | 0 (0) | 0 (0) | 0 (0) | 0 (0) |
| **cGvHD joints & muscles** | 12 (10.2) | 0 (0) | 11 (25.6) | 1 (7.1) |
| joints mild | 6 (5.1) | 0 (0) | 6 (14.0) | 0 (0) |
| joints moderate | 3 (2.5) | 0 (0) | 3 (7.0) | 0 (0) |
| joints severe | 3 (2.5) | 0 (0) | 2 (4.7) | 1 (7.1) |
| **cGvHD lung** | 15 (12.7) | 0 (0) | 14 (32.6) | 1 (7.1) |
| lung mild | 6 (5.1) | 0 (0) | 6 (14.0) | 0 (0) |
| lung moderate | 7 (5.9) | 0 (0) | 6 (14.0) | 1 (7.1) |
| lung severe | 2 (1.7) | 0 (0) | 2 (4.7) | 0 (0) |
| **cGvHD liver** | 14 (11.9) | 0 (0) | 10 (23.3) | 4 (28.6) |
| liver mild | 5 (4.2) | 0 (0) | 5 (11.6) | 0 (0) |
| liver moderate | 9 (7.6) | 0 (0) | 5 (11.6) | 4 (28.6) |
| liver severe | 0 (0) | 0 (0) | 0 (0) | 0 (0) |
| **cGvHD other organs** | 16 (13.6) | 0 (0) | 14 (32.6) | 2 (14.3) |
| Abbreviations: upperGI = upper gastrointestinal tract, lowerGI / loGI = lower gastrointestinal tract, GI = gastrointestinal tract | | | | |

**Table S3** **Flares of GvHD in aGVHD, classical cGVHD and overlap cGVHD**

|  | **overall** | **acute** | **classical chronic** | **overlap chronic** |
| --- | --- | --- | --- | --- |
| n (%) | 118 | 61 | 43 | 14 |
| **flare of known GvHD while RUX treatment** | | | | |
| **yes** | 50 (42.4) | 30 (49.2) | 14 (32.6) | 6 (42.9) |
| **chronic GvHD intermittently present under RUX treatment** | | | | |
|  | 56 (47.5) | 21 (34.4) | 25 (58.1) | 10 (71.4) |
| **new onset of GvHD organs not affected prior RUX initiation** | | | | |
| **no new onset** | 73 (61.9) | 35 (57.4) | 31 (72.1) | 7 (50.0) |
| **overall** | 45 (38.1) | 26 (42.6) | 12 (27.9) | 7 (50.0) |
| **aGvHD** | 8 (6.8) | 8 (13.1) | 0 (0) | 0 (0) |
| aGvHD upperGI | 0 (0) | 0 (0) | 0 (0) | 0 (0) |
| aGvHD lowerGI | 3 (2.5) | 3 (4.9) | 0 (0) | 0 (0) |
| aGvHD skin | 6 (5.1) | 6 (9.8) | 0 (0) | 0 (0) |
| aGvHD liver | 0 (0) | 0 (0) | 0 (0) | 0 (0) |
| **cGvHD** | 40 (33.9) | 21 (34.4) | 12 (27.9) | 7 (50.0) |
| cGvHD skin | 17 (14.4) | 8 (13.1) | 3 (7.0) | 6 |
| cGvHD GI | 3 (2.5) | 2 (3.3) | 1 (2.3) | 0 (0) |
| cGvHD eyes | 17 (14.4) | 7 (11.5) | 8 (18.6) | 2 (14.3) |
| cGvHD mouth | 18 (15.3) | 14 (23.0) | 2 (4.7) | 2 (14.3) |
| cGvHD joints | 7 (5.9) | 4 (6.6) | 1 (2.3) | 2 (14.3) |
| cGvHD lung | 1 (0.9) | 0 (0) | 1 (2.3) | 0 (0) |
| cGvHD liver | 4 (3.4) | 4 (6.6) | 0 (0) | 0 (0) |
| cGvHD other | 0 (0) | 0 (0) | 0 (0) | 0 (0) |

In this table, reoccurrence of GvHD in organs described at first RUX dose that had responded in the meantime and new presentation of GvHD in prior unaffected organs are mentioned separately.

**Table S4** **Organs difficult to treat (cGvHD lung/skin)**

| **Chronic GvHD of the lung at RUX initiation (n=15)** | |
| --- | --- |
| stage 1 | 6 |
| stage 2 | 7 |
| stage 3 | 2 |
| response in lung cGvHD | |
| CR | 2 (both initially st2) |
| PR | 2 (both initially st2) |
| SD | 9 (n=4 initially st1, n=3 initially st2, n=2 initially st3) |
| PD | 2 (both initially st1) |

**Table S5** **GvHD treatment at last follow up in aGVHD, classical cGVHD and overlap cGVHD**

|  | **overall** | **acute** | **classical chronic** | **overlap chronic** |
| --- | --- | --- | --- | --- |
| n (%) | 118 | 61 | 43 | 14 |
| **RUX discontinued** | 69 (58.5) | 45 (73.8) | 18 (41.9) | 6 (42.9) |
| **RUX ongoing at last follow up** | 49 (41.5) | 16 (26.2) | 25 (58.1) | 8 (57.1) |
| **ongoing GvHD treatment at last follow up**, n (% of overall cohort) | | | | |
| **RUX monotherapy** | 24 (20.3) | 9 (14.8) | 10 (23.3) | 5 (35.7) |
| **RUX + steroids** | 8 (6.8) | 3 (4.9) | 4 (9.3) | 1 (7.1) |
| **RUX + agent(s) other than steroids** | 17 (14.4) | 4 (6.6) | 11 (25.6) | 2 (14.3) |

**Table S6 Causes of death in aGVHD, classical cGVHD and overlap cGVHD**

| n (%*) | **overall** | **acute** | **classical chronic** | **overlap** |
| --- | --- | --- | --- | --- |
|  | 118 | 61 | 43 | 14 |
| **dead** | 38 (32.2) | 26 (42.6) | 9 (20.9) | 3 (21.3) |
| **causes of death** | | | | |
| **relapse** | 8 (21.1) | 4 (15.4) | 3 (33.3) | 1 (33.3) |
| **relapse + GvHD** | 1 (2.6) | 1 (3.8) | 0 (0) | 0 (0) |
| **GvHD** without infection | 8 (21.1) | 5 (19.2) | 2 (22.2) | 1 (33.3) |
| **GvHD with infection** | 9 (23.7) | 7 (26.9) | 2 (22.2) | 0 (0) |
| **infection** without GvHD | 8 (21.1) | 5 (19.2) | 2 (22.2) | 1 (33.3) |
| **secondary malignancy** | 1 (2.6) | 1 (3.8) | 0 (0) | 0 (0) |
| **other non-relapse causes** | 3 (7.9) | 3 (11.5) | 0 (0) | 0 (0) |
| *Percentage of causes of death was calculated in relation to overall death rate | | | | |

**Table S7** **aGVHD, classical cGVHD and overlap cGVHD characteristics at RUX initiation**

|  | **overall** | **acute** | **classical chronic** | **overlap chronic** |
| --- | --- | --- | --- | --- |
|  | 118 | 61 | 43 | 14 |
| **time from transplantation to RUX initiation in months** | | | | |
| median [range] | 7.0  [0.3 to 170.2] | 1.5  [0.3 to 72.0] | 23.4  [5.5 to 170.2] | 9.1  [2.4 to 64.0] |
| **acute GvHD overall severity at RUX initiation**, n (%) | | | | |
| **no aGvHD** | 45 (38.1) | 0 (0) | 43 (100) | 2 (14.3) |
| **aGvHD grade 1** | 7 (5.9) | 0 (0) | 0 (0) | 7 (50.0) |
| **aGvHD grade 2** | 15 (12.7) | 13 (21.3) | 0 (0) | 2 (14.3) |
| **aGvHD grade 3** | 44 (37.3) | 42 (68.9) | 0 (0) | 2 (14.3) |
| **aGvHD grade 4** | 7 (5.9) | 6 (9.8) | 0 (0) | 1 (7.1) |
| **chronic GvHD overall severity at RUX initiation**, n (%) | | | | |
| **no cGvHD** | 61 (51.7) | 61 (100) | 0 (0) | 0 (0) |
| **cGvHD mild** | 6 (5.1) | 0 (0) | 3 (7.0) | 3 (21.4) |
| **cGvHD moderate** | 27 (22.9) | 0 (0) | 19 (44.2) | 8 (57.1) |
| **cGvHD severe** | 24 (20.3) | 0 (0) | 21 (48.8) | 3 (21.4) |
| **steroid status**, n (% of overall cohort) | | | | |
| **primary steroid refractory*** | 21 (17.8) | 17 (27.9) | 2 (4.7) | 2 (14.3) |
| **steroid dependent (failed taper)*** | 12 (10.2) | 5 (8.2) | 4 (9.3) | 3 (21.4) |
| **steroid-refractory criteria not met**** | 52 (44.1) | 23 (37.7) | 22 (51.2) | 7 (50.0) |
| **RUX beyond second/third line***** | 33 (28.0) | 16 (26.2) | 15 (34.9) | 2 (14.3) |
| GvHD Grading was performed according to criteria mentioned below.  * RUX was used in 2nd line (aGVHD/overlap) or 2^nd^/3^rd^ line (classical cGVHD), and steroid dependent/refractory according to REACH study inclusion criteria.[18,49,50]  ** RUX was used in 2nd line (aGVHD/overlap) or 2^nd^/3^rd^ line (classical cGVHD), but the stringent refractory/dependency criteria as above were not met due to insufficient steroid exposure (dosing and/or duration).  *** RUX was used > 2nd line (acute GVHD and overlap) or >3rd (classical cGVHD), and previous line(s) were ineffective or intolerant. | | | | |
