# Supplementary material for: Long-term follow-up of ruxolitinib pre-approval use in acute and chronic graft-versus-host disease
Source: Ann Hematol. 2026 Aug 6;105(8):358. doi: 10.1007/s00277-026-07217-4 (PMC13447468; doi:10.1007/s00277-026-07217-4)
Supplement: Supplementary file 2 [file 277_2026_7217_MOESM2_ESM.docx]

**Supplementary material figures**

**Fig. S1** **GvHD transition in patients with acute GvHD**

Chronic GVHD I = NIH cGVHD mild, cGVHD II = cGVHD NIH moderate

**Fig. S2** **GvHD transition in patients with classical chronic GvHD**

cGVHD I = NIH cGVHD mild, cGVHD II = cGVHD NIH moderate, cGVHD III = cGVHD NIH severe

**Fig. S3a Best response in overlap cGvHD**

“RUX in combination” is including patients responding to RUX alone and those responding after the addition of another treatment to RUX.

**Fig. S3b** **Response in overlap cGvHD at 1 month, 3 months, 6 months and last follow-up**

*Response rates were evaluated at defined time points, regardless of whether RUX was used alone or in combination

*No response= Stable disease, mixed response, progressive disease
